# Climatic suitability screening of *Parthenocissus tricuspidata* and *Ficus tikoua* as candidate lianas for rocky slope rehabilitation

**DOI:** 10.3389/fpls.2026.1790833

**Published:** 2026-04-13

**Authors:** Zhengfeng Chen, Hong Li, Xing Wen, Lianghua Huang, Li Chen, Wenguo Wang

**Affiliations:** 1 China Southwest Geotechnical Investigation & Design Institute Co. Ltd., Chengdu, Sichuan, China; 2 Wildlife Forensic Science Service, Kunming, China; 3 Biogas Institute of Ministry of Agriculture and Rural Affairs, Chinese Academy of Agricultural Sciences, Chengdu, China

**Keywords:** climatic niche, liana, MaxEnt, remediation, rocky slope

## Abstract

**Introduction:**

Infrastructure development in karst regions frequently leads to the formation of rocky slopes that are difficult to rehabilitate. Lianas, owing to their strong adaptability and rapid growth, may provide suitable plant materials for the ecological rehabilitation of such slopes. Among them, *Parthenocissus tricuspidata* and *Ficus tikoua* are two common liana species with traits that may support rocky slope rehabilitation, but their broad-scale climatic suitability remains unclear.

**Methods:**

In this study, the MaxEnt model was used to predict the global climatically suitable areas of *P. tricuspidata* and *F. tikoua* under current and future climate scenarios. The potential distributions of both species were compared to evaluate their suitability as candidate species for rocky slope rehabilitation.

**Results:**

Under current climatic conditions, climatically suitable areas for both species, including overlapping suitable regions, were mainly distributed in parts of North America, East and Southeast Asia, Eastern Australia, and Central Europe. Under the selected future climate scenarios, the climatically suitable areas of both species generally increased, although the magnitude and pattern of change differed between species and scenarios.

**Discussion:**

These results provide a broad-scale climatic screening framework for evaluating *P. tricuspidata* and *F. tikoua* as candidate species for rocky slope rehabilitation. However, the predicted suitable areas represent climatic suitability rather than direct restoration site identification. Practical application should therefore also consider real-world restoration constraints, including rocky slope occurrence, substrate conditions, and ecological risk assessment, especially in non-native regions.

## 1 Introduction

Transportation construction (e.g., construction of highways and railroads) and mining activities can lead to exposed rocky slopes, causing not only damage to local vegetation, soil, and ecological landscapes but also inducing soil erosion and related geological disasters (Chang et al., 2022; Lee et al., 2007). Ecological rehabilitation remains the main approach to prevent these serious environmental issues. Karst regions, characterized by their abundant rock slopes, are globally widespread. These areas cover 15.2% of the world’s ice-free continental surface, with Europe exhibiting the highest proportional coverage (21.8%) and Asia possessing the largest absolute area (8.35 million km²) (Goldscheider et al., 2020). Globally, 31.1% of carbonate rock exposures occur in plains, while approximately 34.2% are subject to arid climates. Notably, 1.18 billion people (16.5% of the global population) inhabit karst regions, with Asia containing the highest absolute population in such regions (661.7 million), whereas Europe (25.3%) and North America (23.5%) demonstrate the greatest proportional representations within them (Goldscheider et al., 2020). However, human activities require considerable infrastructure support. Infrastructure development in karst zones poses substantial environmental risks, particularly through the formation of exposed rock slopes. These disturbed rocky slopes are often difficult to restore because of shallow substrates, high rock exposure, low water-holding capacity, and strong microhabitat heterogeneity, all of which constrain plant establishment and long-term slope stability.

Traditional rocky slope protection methods (e.g., cement plastering, shotcrete spray, and mortar rubble) are not particularly beneficial to the local ecology and environment (Matsubara, 2021). Therefore, it is important to develop and implement novel methods that can actually rehabilitate exposed rocky slopes. The rehabilitation of rocky slopes is often conducted using artificial soil layers or ecological substrate layers in combination with herbs and/or shrubs (Li et al., 2018; Peng et al., 2021). Vine and liana species demonstrate adaptability and growth capacity in karst-dominated rocky slope environments (Xing and Wu, 2014; Zhang et al., 2023). In addition, such approaches also include the use of free-standing planted gabions (Beikircher et al., 2010), combining plant species selection, soil improvement techniques, and surface texturing technology (Wang et al., 2021). The selection of suitable plant species is crucial to ensure the effective rehabilitation of rock slopes. Herbs and small shrubs have been extensively used in most contemporary rehabilitation projects (Peng et al., 2021). However, restoration success is not determined by plant presence alone; it also depends on the interaction between plant traits and real world site constraints, such as slope aspect, lithology, fissure conditions, substrate thickness, and soil water and nutrient availability. Therefore, plant selection for rocky slope rehabilitation requires not only ecological suitability but also consideration of practical restoration constraints.

Generally, lianas are characterized by their high growth rates, high resistance to drought stress, high soil consolidation efficiency, and rapid canopy formations, making them ideal plants for enhanced vegetation-based rehabilitation and landscaping (Liu et al., 2011; Pérez et al., 2021; Yang et al., 2018; Zhou et al., 2022). Adhesive climbers with suckers or aerial roots offer unique benefits for the ecological rehabilitation processes of rocky slopes. Therefore, analyzing the distribution patterns of liana species under current and future climatic conditions can provide critical guidance for determining their applicable scope in the ecological rehabilitation of rocky slopes. Nevertheless, climatic suitability alone cannot identify actual restoration sites, because the practical use of species in rocky-slope rehabilitation also depends on whether suitable rocky or karst habitats are truly present and whether local ecological and engineering conditions permit successful establishment. Thus, species distribution modelling should be regarded as a first-step screening approach rather than a direct substitute for site level restoration design.

*Parthenocissus tricuspidata*, which has tendrils, and *Ficus tikoua*, which has adventitious roots, are liana species belonging to Vitaceae and Moraceae, respectively. Both species possess attachment strategies that make them potentially valuable for slope revegetation, but their ecological requirements and restoration functions may differ. *P. tricuspidata* can climb on different substrates, including rocky slopes and building facades, owing to its adhesive discs, this ability enables the plant to resist strong winds and storms (He et al., 2011). Additionally, *P. tricuspidata* can effectively adapt to water-scarce environments by regulating its biomass allocation, nutrient uptake, and nutrient use efficiencies (Wang et al., 2009). On the other hand, *F. tikoua* can climb on rocks and tree trunks, and it also has strong resistance to extreme environmental conditions, including droughts. Indeed, *F*. *tikoua* can effectively grow in wastelands, grasslands, sandy slopes, and rock crevices. The specific physiological characteristics and habits of *P. tricuspidata* and *F. tikoua* suggest great potential of these plant species in the ecological rehabilitation of rocky slopes. However, previous relevant studies on these plant species have focused primarily on their physiological characteristics. In addition, although both species may be promising candidates for rehabilitation, their broad scale climatic suitability should not be automatically interpreted as management suitability, especially outside their native or ecologically compatible regions, where ecological risk and potential invasive behavior must also be considered. Therefore, there is still a critical need to ascertain the current research status of these plants, predict their distribution and dynamics under current and future climate conditions, and further develop their research and development.

This study uses MaxEnt as a climate based screening tool to assess the current and future potential suitability of *P. tricuspidata* and *F. tikoua* and to explore their potential as candidate species for rocky slope ecological rehabilitation at a broad regional scale. More specifically, it aims to identify broad climatically suitable regions for the two liana species, compare the main environmental factors shaping their distributions, and provide a preliminary basis for candidate species screening in rocky slope rehabilitation. The results are intended to support broad scale species screening and regional prioritization, rather than to directly delineate restoration implementation sites or prescribe site specific restoration strategies.

## 2 Materials and methods

### 2.1 Studied species

*Parthenocissus tricuspidata* is a large, woody, deciduous liana primarily distributed across Asia (Nie et al., 2010). In the wild, the plant is distributed along cliff walls and in shrublands of mountain slopes at altitudes of 150–1200 m. This species is widely cultivated as an attractive ornamental climber in China and many other countries. However, data from the USDA PLANTS Database (https://plants.usda.gov/home) and literature surveys indicate that despite its presence in North America, Canada, and some European countries, it is not indigenous to these regions but rather a non-native species introduced locally as a rock-climbing ornamental plant (Burda and Koniakin, 2019; Frappier and Eckert, 2003; Nie et al., 2010).

*Ficus tikoua* is a creeping woody liana with slender adventitious roots on its stem. The species is primarily distributed in China, northeastern India, northern Vietnam, and Laos within Asia (Kuo et al., 2017; Zhang et al., 2023). In the wild, the species grows in sparse forests, grassy slopes, and rocky crevices in low-elevation mountain areas. Furthermore, its fruit is edible, and it is an excellent soil and water conservation plant. Compared with *P. tricuspidata*, *F. tikoua* has a more geographically restricted known distribution, which should be taken into account when interpreting broad scale modelling results.

**Figure 1:**
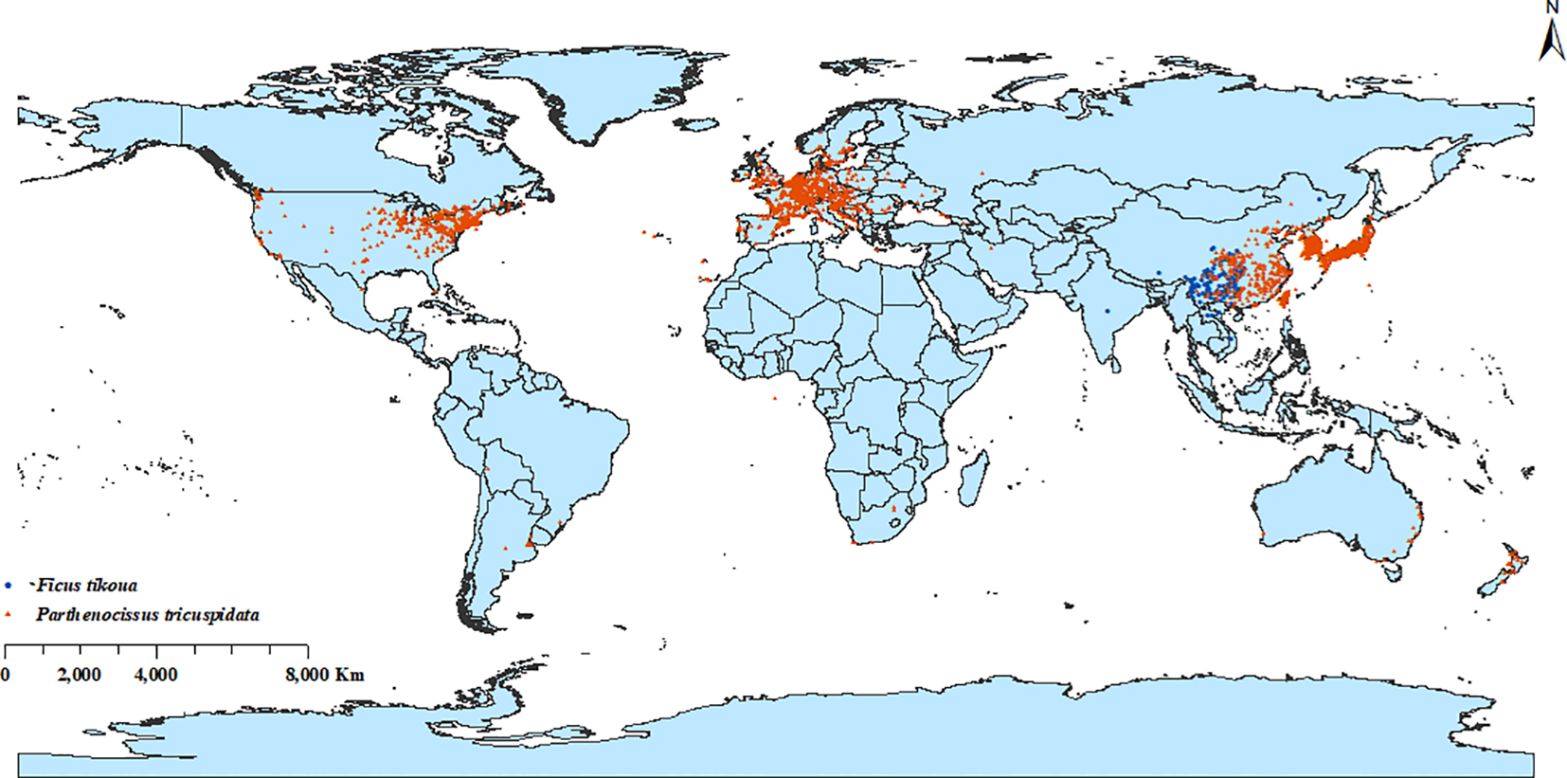
Spatial distributions of *Parthenocissus tricuspidata* (red dots) and *Ficus tikoua* (blue dots) globally.

Occurrence records representing the known distribution ranges of *P. tricuspidata* and *F. tikoua* were compiled from the Global Biodiversity Information Facility (http://www.gbif.org; accessed on 26 May 2025). To improve data quality, records with missing coordinates, obvious geographic errors, duplicates, and taxonomically doubtful entries were removed before modelling. Spatial thinning of occurrence records was performed using the R package spThin to remove redundant records occurring within a 10-km distance threshold (Aiello-Lammens et al., 2015). This thinning step was used to reduce spatial clustering and sampling bias in the occurrence data. In total, 2947 records for *P. tricuspidata* and 141 for *F. tikoua* were selected for MaxEnt modeling (**Figure 1**). Due to the relatively limited number of records of the occurrence of *F. tikoua* retained among them, the model output should be interpreted as a large scale climate suitability screening rather than the final representation of its global distribution.

### 2.2 Climate data

Current climatic data with a 2.5-min resolution, including 19 climate variables corresponding to the current climate conditions (1970–2000), were downloaded from the WorldClim database released in January 2020 (https://worldclim.org/) (**Table 1**) (Brown et al., 2018; Hijmans et al., 2005). Future climate projections were derived from the BCC-CSM2-MR global climate model under two shared socio-economic pathways (SSP126 and SSP585) for the periods 2021–2040, 2041–2060, and 2061–2080 based on CMIP6 downscaled datasets. The model was selected due to its reliable simulation of key climatic variables, its widespread application in climate change impact assessments, and its provide a consistent climate-forcing framework for broad-scale screening. Although multi-model ensembles may better capture climate uncertainty, the use of a well-established single GCM remains a common practice in species distribution modeling.

**Table 1:** Bioclimatic and soil variables used in prediction modelling.

Variable	Description	Variables	Description
BIO1	Mean Annual Temperature	BIO15	Precipitation Seasonality (Coefficient of Variation)
BIO2	Mean Diurnal Range	BIO16	Precipitation in the Wettest Quarter
BIO3	Isothermality (BIO2/BIO7) (×100)	BIO17	Precipitation in the Driest Quarter
BIO4	Temperature Seasonality	BIO18	Precipitation in the Warmest Quarter
BIO5	Max Temperature of the Warmest Month	BIO19	Precipitation in the Coldest Quarter
BIO6	Min Temperature in the Coldest Month	AWC_CLASS	Available Water Capacity Class
BIO7	Annual Temperature Range (BIO5-BIO6)	REF_DEPTH	Reference Depth
BIO8	Mean Temperature in the Wettest Quarter	T_CACO3	Total Calcium Carbonate
BIO9	Mean Temperature in the Driest Quarter	T_CASO4	Total Calcium Sulfate
BIO10	Mean Temperature in the Warmest Quarter	T_CLAY	Total Clay Content
BIO11	Mean Temperature in the Coldest Quarter	T_ECE	Total Exchangeable Cations
BIO12	Annual Precipitation	T_GRAVEL	Total Gravel Content
BIO13	Precipitation in the Wettest Month	T_OC	Total Organic Carbon
BIO14	Precipitation in the Driest Month	T_PH_H2O	pH in Water

Furthermore, soil data were acquired from the Harmonized World Soil Database (https://www.fao.org/soils-portal/soil-survey/soil-maps-and-databases/harmonized-world-soil-database-v12/en/). Nine parameters were extracted from this database: soil available water capacity (AWC_CLASS), calcium carbonate or lime content (T_CACO), soil reference depth (REF_DEPTH), gravel volume percentage (T_GRAVEL), sulfate content (T_CASO4), clay content (T_CLAY), electrical conductivity (T_ECE), organic carbon content (T_OC), and soil pH in water (T_PH_H2O) (**Table 1**). Soil scientific variables in karst topography have significant ecological significance for substrate conditions and rock slope restoration. However, the contribution of these variables to the model’s interpretation is expected to vary by species rather than be the same across all models. The world map data utilized in this study were sourced from the Resource and Environmental Science and Data Center (RESDC) of the Institute of Geographic Sciences and Natural Resources Research, Chinese Academy of Sciences (https://www.resdc.cn/) [Map Review No.: GS(2016)1667]. These data provided a unified spatial framework for the global distribution analysis of *P tricuspidata* and *F*. *tikoua*, as well as their associated environmental variables.

### 2.3 Prediction of the potential habitats of *P. tricuspidata* and *F. tikoua*

#### 2.3.1 Screening of the main environmental variables

High correlations may exist among environmental variables, which can lead to model overfitting, consequently compromising both model accuracy and generalization capability. To address this issue, the present study employed ENMTools 1.4 software to calculate the pairwise correlations among environmental variables. Variables exhibiting a Pearson correlation coefficient (PCC) absolute value greater than 0.8 were identified as highly correlated. Preliminary MaxEnt runs were subsequently conducted to evaluate the relative contribution of each predictor. When strong correlations were identified, variables with lower contributions within correlated pairs were excluded, whereas those with greater ecological relevance were retained to optimize model inputs (Zhang et al., 2024). This procedure was intended to reduce multicollinearity while retaining variables that were both informative and ecologically meaningful. In assessing broad-scale climatic suitability for candidate rocky-slope rehabilitation areas, predictor selection emphasized not only statistical performance but also ecological relevance to plant establishment, particularly under climatic stress and substrate-related conditions. This two-step procedure reduced redundancy among predictors and generated a parsimonious set of environmental variables for subsequent modeling.

#### 2.3.2 Optimization of MaxEnt model parameters

The MaxEnt model is widely used in datasets that only contain information on the emergence of species and performs well when the sample size is limited. Further optimize the model complexity by using the kuenm package to reduce overfitting and improve the interpretability of the model. During this process, the kuenm package was used to optimize the feature classes (FC) and regularization multipliers (RM) for *P. tricuspidata* and *F. tikoua*, with the aim of improving predictive accuracy and the reliability of the model results (Tamura et al., 2011).

The RM values were set to range from 0.1 to 4.0, with an increment of 0.1. A total of 31 combinations of five feature classes—linear (L), quadratic (Q), hinge (H), product (P), and threshold (T)—were tested. These combinations included: L, Q, P, T, H, LQ, LP, LT, LH, QP, QT, QH, PT, PH, TH, LQP, LQT, LQH, LPT, LPH, LTH, QPT, QPH, QTH, PTH, LQPT, LQPH, LQTH, LPTH, QPTH, and LQPTH. The Akaike information criterion corrected for small sample sizes (AICc) was used to evaluate model fit and complexity across different parameter combinations (Warren and Seifert, 2011; Cobos et al., 2019). The area under the receiver operating characteristic curve (AUC) was applied to assess the ability of each parameter combination to discriminate between test occurrence points and background points. In addition, model performance was evaluated using the difference between training and testing AUC values, the 5% training omission rate, and the Minimum Training Presence omission rate. Based on these criteria, the parameter combination most suitable for species distribution modeling was selected (Giraldo and Kucuker, 2024).

#### 2.3.3 Prediction of the potential distribution of *P. tricuspidata* and *F. tikoua* using the MaxEnt model

The spatial distribution points of *P. tricuspidata* and *F. tikoua*, as well as the selected environmental data, were imported into the MaxEnt software. MaxEnt randomly selected 75% and 25% of the distribution data for modeling training and testing, respectively, with 10 replications. MaxEnt modeling was performed using a maximum of 5000 iterations with 10 simulations, and the average value of 10 simulations was used as the result. Linear, quadratic, and hinge features were used for model training (Shi et al., 2024). The results were output as logistic format data in ASCII files. The obtained feature results were further converted into raster data in ArcGIS software (Phillips et al., 2006, 2017).

The MaxEnt prediction results obtained in this study were imported into ArcGIS software in raster format and overlaid onto a global vector basemap. The global distribution areas of *P. tricuspidata* and *F. tikoua* were classified into four suitability categories: unsuitable areas, poorly suitable areas, moderately suitable areas, and highly suitable areas. The predicted suitable habitats were classified using the Natural Breaks (Jenks) classification method within ArcGIS software (Zhang et al., 2021). The classification criteria of the MaxEnt-based distribution areas were mainly derived from the survival probability (*p*) of each plant species, including unsuitable areas (*p* ≤ 0.008), poorly suitable areas (0.008 < *p* ≤ 0.25), moderately suitable areas (0.25 < *p* ≤ 0.47), and highly suitable areas (*p* > 0.47) (Huang et al., 2020). Meanwhile, areas of each level of distribution suitability and the overall potential distribution were calculated. In addition, SDMtoolbox (http://www.sdmtoolbox.org/) in ArcGIS 10.8 was used to analyze the centroid position and its migration trend for both *P. tricuspidata* and *F. tikoua* potential distributions under the different future climate scenarios, respectively.

#### 2.3.4 Evaluation of the MaxEnt model prediction results

The AUC of the receiver operating characteristic (ROC) curve was used to evaluate the accuracy of the model. The AUC value can vary between 0 and 1. Values close to 1 indicate that there is no overlap between the presence and pseudo-absence of the species and that the two were well discriminated (Abay and Gül, 2024; Zank et al., 2014). In addition, response curves were examined to identify environmentally favorable conditions for the major contributing variables, with predictor intervals associated with relatively high logistic suitability values used to characterize suitable environmental ranges (Washaya et al., 2025).

## 3 Results

### 3.1 Environmental variable screening

**Figure 2:**
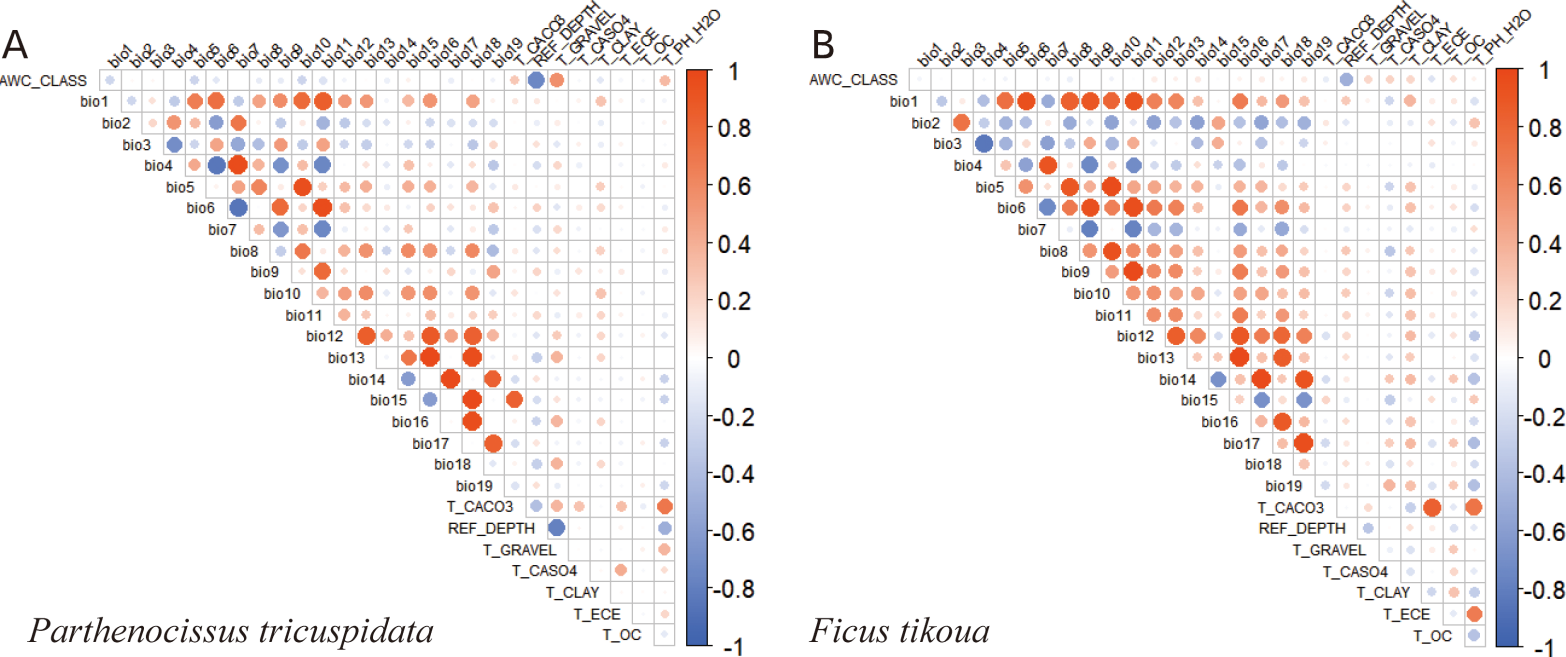
Correlations between the environmental variables of distribution data for *Parthenocissus tricuspidata* **(A)** and *Ficus tikoua* **(B)**. The red and blue colors indicate positive and negative correlations, respectively. The darker the color, the closer the value is to 1 or -1, respectively, indicating stronger correlations.

A subset of environmental variables was retained for modeling after removing highly correlated predictors (**Supplementary Table 1**; **Figure 2**). For *P. tricuspidata*, precipitation of the driest quarter, annual precipitation, annual mean temperature, mean temperature of the coldest quarter, and temperature seasonality each contributed more than 10% to the model. In contrast, the distribution of *F. tikoua* was primarily influenced by precipitation of the warmest quarter, temperature seasonality, and precipitation seasonality, all of which showed relative contributions exceeding 10%. The final model incorporated 11 environmental variables for each species. For *P. tricuspidata*, the most influential predictors included mean diurnal range (bio2), isothermality (bio3), temperature seasonality (bio4), maximum temperature of the warmest month (bio5), mean temperature of the driest quarter (bio9), mean temperature of the coldest quarter (bio11), annual precipitation (bio12), precipitation seasonality (bio15), precipitation of the driest quarter (bio17), total gravel content (T_GRAVEL), and total clay content (T_CLAY). For *F. tikoua*, key predictors included temperature seasonality (bio4), minimum temperature of the coldest month (bio6), precipitation seasonality (bio15), precipitation of the warmest quarter (bio18), precipitation of the coldest quarter (bio19), available water capacity class (AWC_CLASS), reference depth (REF_DEPTH), total gravel content (T_GRAVEL), total calcium sulfate (T_CASO4), total clay content (T_CLAY), and soil pH (T_PH_H2O). Overall, both climatic and soil-related predictors were retained after screening, but their apparent importance differed between the two species. For *P. tricuspidata*, the retained predictor set was dominated by climatic variables, whereas for *F. tikoua*, both climatic and pedological variables contributed to the final environmental profile. This result suggests that substrate related factors may provide greater ecological interpretation for *F. tikoua* than for *P. tricuspidata* at the present modelling scale.

### 3.2 Model optimization

To improve the predictive accuracy and reliability of the MaxEnt models, while avoiding excessive computational burden, the kuenm package was used to model the distribution data of the two plant species in combination with the selected environmental variables. For both *P. tricuspidata* and *F. tikoua*, a total of 1, 240 candidate models were generated, all of which were statistically significant. Based on the delta AICc values and other model evaluation metrics, the optimal parameter configuration for *P. tricuspidata* was identified as a regularization multiplier (RM) of 0.8 with the feature class (FC) combination “LP”. For *F. tikoua*, the optimal model was characterized by an RM value of 1.7 and the feature class combination “QTH” (**Supplementary Table 2**). These parameter combinations were used in the final MaxEnt runs, thereby ensuring consistency between the optimization procedure and the subsequent suitability projections. The optimized settings indicate that the two species differed in the level of model complexity required to adequately capture their broad-scale climatic responses.

### 3.3 Model validation

**Figure 3:**
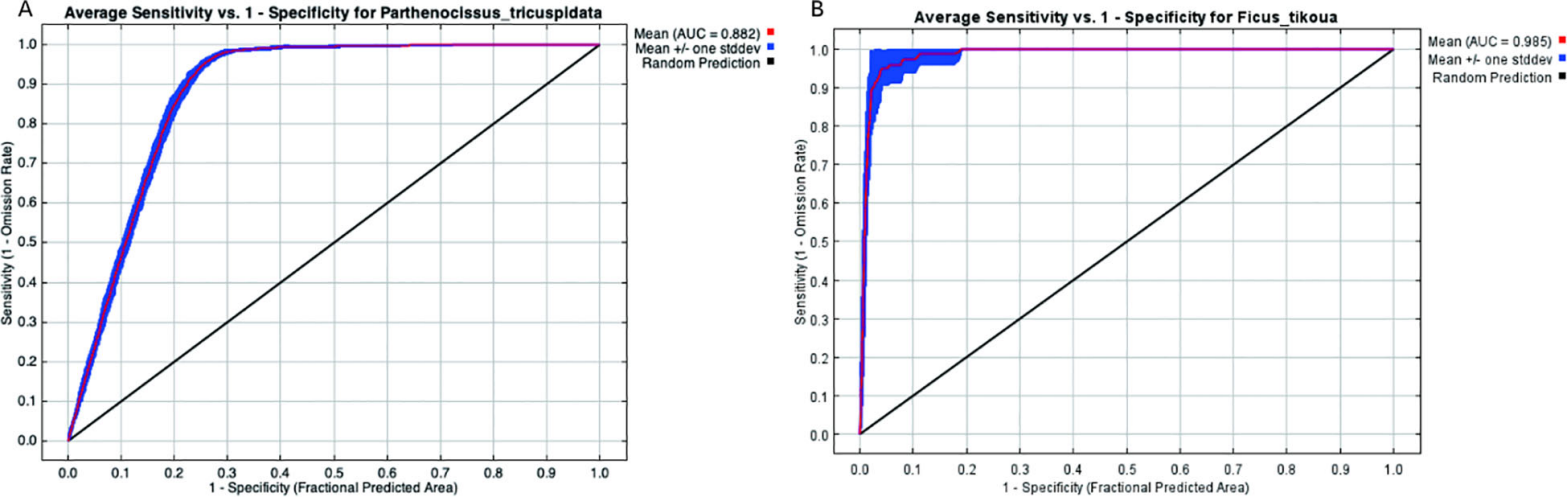
Receiver operating characteristic curve plot of the MaxEnt-based predicted suitable areas for *Parthenocissus tricuspidata* **(A)** and *Ficus tikoua* **(B)**.

The ROC curve was used to assess the accuracy of the MaxEnt-based predicted results of the suitable habitat areas (**Figure 3**). Under the current climate conditions, the AUC values of the results obtained at the training step were 0.882 and 0.985 for *P. tricuspidata* and *F. tikoua*, respectively. The AUC of the MaxEnt-based predicted results was substantially greater than those of the random prediction distribution model (0.500). The obtained results demonstrated the suitability of the plant distribution sites of *P. tricuspidata* and *F. tikoua* and the environmental variables for predicting suitable habitats for these two species. These results indicate that, within the current modelling framework, the optimized models had a relatively strong ability to discriminate occurrence records from background points. However, because AUC values may be sensitive to model extent, background selection, and the structure of presence-only data, especially for species with relatively limited occurrence records, the present results are more suitable for broad-scale climatic suitability screening than for precise delineation of restoration sites.

### 3.4 Climate variable assessment

The selected environmental variables were used to construct the MaxEnt models with optimized parameter settings. **Table 2** lists the top five environmental variables ranked by their relative contributions to the predicted potential distribution models of *P. tricuspidata* and *F. tikoua*.

**Table 2:** The environmental variables in terms of contribution rate to the distribution range of *Parthenocissus tricuspidata* and *Ficus tikoua*.

Species	Variable	Percent contribution	Permutation importance	Suitable range
*Parthenocissus tricuspidata*	bio4	26.3	25.5	716.531 - 2579.135
	bio11	24.5	35.5	0.101 - 36.093 °C
	bio9	16.5	6	-6.832 - 12.856 °C
	bio12	14.6	2.9	640.126 - 1909.530 mm
	bio2	5	13.8	7.601 -10.226 °C
	bio3	4	6.1	27.906 -109.012
	bio17	4	0.6	-141.3 - 357.206 mm
	bio15	2.4	1.8	6.076 - 66.667
	bio5	2.3	7.5	21.130 -31.068 °C
	T_GRAVEL	0.2	0.2	0.056 – 77
	T_CLAY	0.1	0.1	12.970 - 92.400
*Ficus tikoua*	bio18	35.7	6.5	464.736 - 2719.200 mm
	AWC_CLAS	25.6	25.5	{1, 5}
	bio19	13.2	12.5	-419.300 - 83.860 mm
	T_CACO_3_	8.9	11.4	0.012 - 0.121 g·kg^-1^
	T_CLAY	5.5	1.9	22.042 - 92.400%
	bio6	3.8	30.2	-0.078 - 31.382 °C
	bio4	2.5	10	600.907 - 2590.117 °C
	bio15	2	0.4	-19.822 - 101.964
	T_GRAVEL	1.7	0.9	2.240 - 77.000%
	T_PH_H_2_O	0.9	0.3	4.899 - 10.480
	REF_DEPTH	0.3	0.4	1.000 - 109.000 cm

The three variables with the highest contribution rate to the current distribution of *P. tricuspidata* were temperature seasonality (bio4), mean temperature in the coldest quarter (bio11), and mean temperature in the driest quarter (bio9), with a cumulative contribution rate of 67.3% and bio 4 having the highest contribution rate of 26.3%. The three variables with the highest contribution rate to the current potential distribution of *F. tikoua* were precipitation of the warmest quarter (bio18), available water capacity class (AWC_CLAS), and mean temperature in the driest quarter (bio9), with a cumulative contribution rate of 74.5% and bio18 having the highest contribution rate of 35.7%. Accordingly, temperature has a greater impact on the potential distribution of *P. tricuspidata*, while precipitation has a greater impact on the potential distribution of *F. tikoua*. under the current climate conditions. In addition, the relatively high contribution of soil available water capacity (AWC_CLASS) in the model for *F. tikoua* indicates that soil water-holding characteristics may have important ecological significance for this species. In contrast, the contribution of soil variables to the model for *P. tricuspidata* was relatively limited, suggesting that its broad-scale potential distribution is more strongly constrained by climatic factors than by the pedological predictors included in this study.

### 3.5 Current potential distribution of *P. tricuspidata* and *F. tikoua*

**Figure 4:**
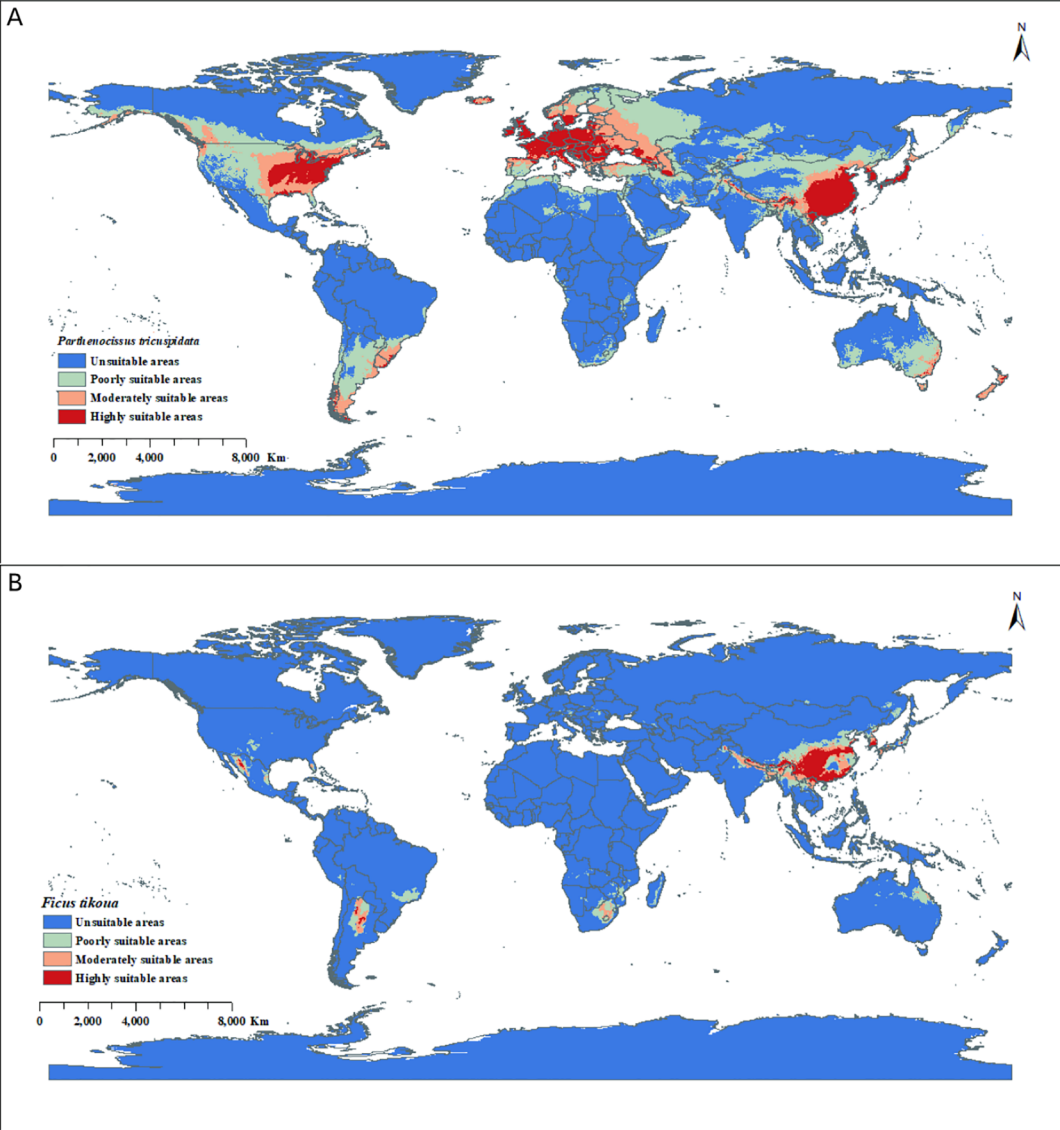
Potential global distribution *Parthenocissus tricuspidata* **(A)** and *Ficus tikoua* **(B)** under current climate conditions as predicted by MaxEnt.

The potential suitable areas for *P. tricuspidata* and *F. tikoua* are shown in **Figure 4**. In this study, potential distribution area refers to the total climatically suitable area, including poorly suitable, moderately suitable, and highly suitable classes. Specifically, the total potential distribution area of *P. tricuspidata* was 49.093 million km^2^. Among this area, the total area of highly suitable area was 10.449 million km^2^, which was mainly within eastern North America (United States), Europe, Asia (southeastern China, Japan, South Korea, Laos, and northern Myanmar), eastern Australia, and southeastern South America (including Uruguay) (**Figure 4A**). In contrast, the total potential distribution area of *F. tikoua* was 8.009 million km^2^. The total area of highly suitable area was 1.839 million km^2^, which were mainly distributed in the Americas (northern Argentina and northern Mexico), Africa (northeastern South Africa), Asia (southwestern China, northeastern India, Laos, South Korea, and Japan), and Oceania (northeastern Australia) (**Figure 4B**). Some climatically suitable regions are geographically coincident with areas known to contain major karst landscapes, but the present study does not explicitly quantify this overlap.

### 3.6 Future changes in suitable habitat of *P. tricuspidata* and *F. tikoua*

**Figure 5:**
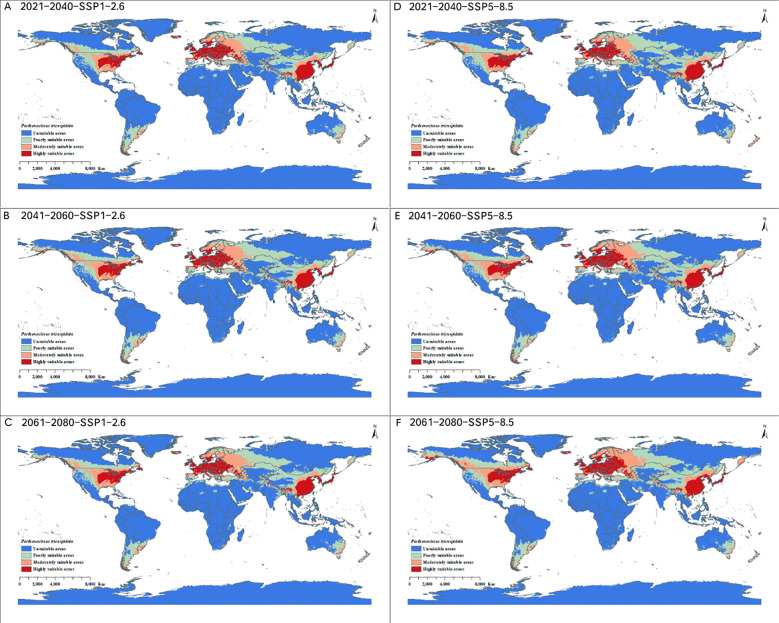
Potential distributions of *Parthenocissus tricuspidata* under **(A)** 2040s SSP1-2.6, **(B)** 2060s SSP1-2.6, **(C)** 2080s SSP1-2.6, **(D)** 2040s SSP5-8.5, **(E)** 2060s SSP5-8.5, and **(F)** 2080s SSP5-8.5 future climate scenarios.

By predicting the potential geographical distribution of *P. tricuspidata* and *F. tikoua* under SSP1-2.6 and SSP5-8.5 climate scenarios in the 2040s, 2060s, and 2080s, we obtained future potential distribution maps for both of these species individually. Meanwhile, the areas of different suitability levels were determined. In the future climate scenarios, potential distribution areas of *P. tricuspidata* and *F. tikoua* generally showed broad changes relative to the current climatic baseline, although the magnitude and direction of change differed between species, periods, and scenarios (**Table 3**). Specifically, Under the SSP1-2.6 and SSP5-8.5 scenarios in the 2040s, the total distribution area of *P. tricuspidata* was predicted to be 51.862 million km^2^ and 53.225 million km^2^, respectively. Among these predicted areas, the highly suitable areas were predicted to increase by 1.489 million km^2^ (14.250%)and 1.914 million km^2^(18.327%), respectively. Under SSP1-2.6 and SSP5-8.5 scenarios in the 2060s, the total distribution area of *P. tricuspidata* was predicted to be 53.390 million km^2^ and 54.789 million km^2^, respectively. Among these areas, the highly suitable areas were predicted to increase by 2.041 million km^2^(19.533%)and 2.820 million km^2^ (26.988%)compared with the current distribution area, respectively. Under SSP1-2.6 and SSP5-8.5 scenarios in the 2080s, the total distribution area of *P. tricuspidata* was 52.287 million km^2^ and 58.680 million km^2^, respectively. Among these areas, the highly suitable areas were predicted to increase by 1.798 million km^2^ (17.207%)and 3.955 million km^2^ (37.851%)compared with the current distribution area, respectively (**Figure 5**).

**Table 3:** Projected suitable habitat areas and change rates for *Parthenocissus tricuspidata* and *Ficus tikoua* under current and future climate scenarios.

Species	Climate model	Period	Poorly suitable areas		Moderately suitable areas		Highly suitable areas		Total distribution area	
			Area (million km^2^)	Rate of change (%)	Area (million km^2^)	Rate of change (%)	Area (million km^2^)	Rate of change (%)	Area (million km^2^)	Rate of change (%)
*Parthenocissus tricuspidata*	–	Current	27.243		11.402		10.449		49.094	
	SSP1-2.6	2040S	27.284	0.150	12.640	10.858	11.938	14.250	51.862	5.638
		2060S	27.220	-0.084	13.680	19.979	12.490	19.533	53.390	8.751
		2080S	26.191	-3.862	13.848	21.452	12.247	17.207	52.287	6.504
	SSP5-8.5	2040S	28.002	2.786	12.859	12.778	12.364	18.327	53.225	8.414
		2060S	26.408	-3.065	15.112	32.538	13.269	26.988	54.789	11.600
		2080S	28.189	3.472	16.087	41.089	14.404	37.851	58.680	19.526
*Ficus tikoua*	–	Current	4.233		1.937		1.839		8.009	
	SSP1-2.6	2040S	8.923	110.796	3.107	60.403	2.384	29.636	14.414	79.973
		2060S	5.415	27.923	2.449	26.433	1.623	-11.746	9.487	18.454
		2080S	7.449	75.974	3.291	69.902	1.873	1.849	12.613	57.485
	SSP5-8.5	2040S	8.063	90.480	3.621	86.939	2.022	9.951	13.707	71.145
		2060S	8.497	100.732	3.192	64.791	2.108	14.628	13.796	72.256
		2080S	7.281	72.006	3.182	64.275	1.768	-3.861	12.231	52.716

**Figure 6:**
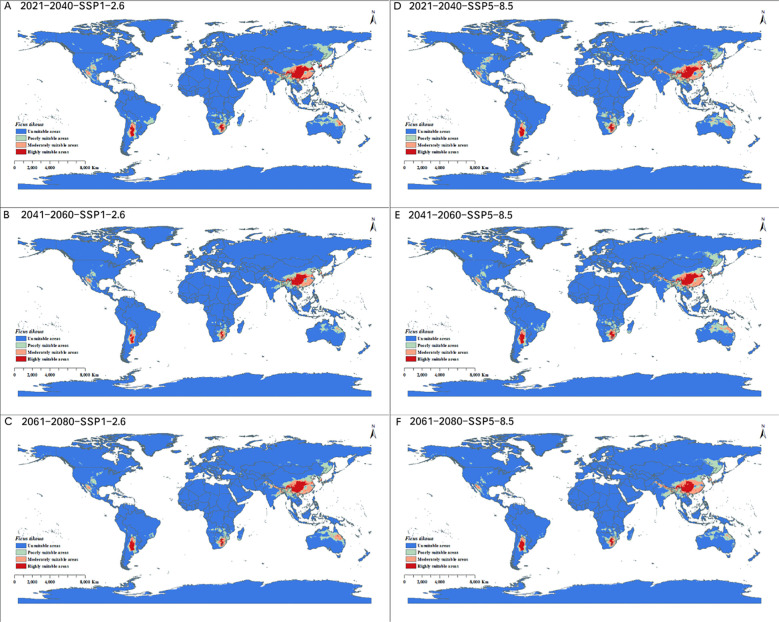
Potential distributions of *Ficus tikoua* under **(A)** 2040s SSP1-2.6, **(B)** 2060s SSP1-2.6, **(C)** 2080s SSP1-2.6, **(D)** 2040s SSP5-8.5, **(E)** 2060s SSP5-8.5, and **(F)** 2080s SSP5-8.5 future climate scenarios.

Under SSP1-2.6 and SSP5-8.5 scenarios in the 2040s, the total distribution area of *F. tikoua* was 14.414 million km^2^ and 13.707 million km^2^, respectively. Among these areas, the highly suitable areas were predicted to increase by 0.545 million km^2^ (29.636%)and 0.183 million km^2^ (9.951%)compared with the current distribution area, respectively.Under SSP1-2.6 and SSP5-8.5 scenarios in the 2060s, the total distribution area of *F. tikoua* was 9.487 million km^2^ and 13.796 million km^2^, respectively. Among these areas, the highly suitable areas were predicted to decrease by 0.215 million km^2^ (-11.746%)under the SSP1-2.6 scenario and increase by 0.269 million km^2^ (14.628%)under the SSP5-8.5 scenario. Under SSP1-2.6 and SSP5-8.5 scenarios in the 2080s, the total distribution area of *F. tikoua* was 12.613 million km^2^ and 12.231 million km^2^, respectively. Among these areas, the highly suitable areas were predicted to increase by 0.035 million km^2^ (1.849%)under the SSP1-2.6 scenario and decrease by 0.071 million km^2^ (-3.861%)under the SSP5-8.5 scenario (**Figure 6**).

Overall, the future projections suggest that the climatically suitable area for *P. tricuspidata* may expand relatively consistently under the selected scenarios, whereas the response of *F. tikoua* appears more variable and scenario dependent. These changes should be interpreted as projected changes in climatic suitability and the spatial redistribution of suitable areas, rather than as direct evidence of ecological adaptation or direct indicators of restoration feasibility. In particular, increased suitability in non-native regions should not be interpreted as a management recommendation, because climatic expansion may also imply increased ecological uncertainty and elevated invasion risk.

## 4 Discussion

### 4.1 Accuracy of MaxEnt model and prediction results

The optimized MaxEnt models demonstrated strong predictive performance for both *P. tricuspidata* and *F. tikoua*, indicating that the modeling framework effectively captured the key environmental factors shaping their potential distributions. The predicted suitable habitats were largely consistent with documented occurrence records, which provides general support for the plausibility of the projected suitability patterns. Comparable levels of predictive accuracy have been widely reported in previous species distribution studies using MaxEnt, further reinforcing the robustness of this approach (Aiello-Lammens et al., 2015; Tamura et al., 2011). At the same time, the present study relied on a single modelling algorithm and a single GCM, and the number of retained occurrence records for *F. tikoua* remained relatively limited for global projection. These features do not invalidate the results, but they meybe limit the robustness and generality of the conclusions. Importantly, the integration of bias-corrected occurrence data with carefully selected environmental variables helped reduce model uncertainty and improved the interpretability of the predictions at a global scale. These projections provide a preliminary climate-based basis for evaluating the potential use of the two liana species in rocky-slope ecological rehabilitation under changing climatic conditions, but they should be complemented by multi-model assessment and site-level validation before practical implementation.

The genus *Parthenocissus* exhibits a disjunct distribution across Asia and North America, with species occurring in both tropical and temperate regions (Nie et al., 2010). The current suitable habitats of *P. tricuspidata* predicted in this study are highly consistent with its documented distribution range. In particular, the species has been widely introduced and successfully established as an ornamental climbing plant in Canada, adjacent regions of the United States, and parts of Europe (Burda and Koniakin, 2019; Frappier and Eckert, 2003; Nie et al., 2010). Given its non-indigenous status, further ecological risk assessments are necessary to evaluate its potential impacts in these regions. *F. tikoua* primarily occurs in China, northeastern India, northern Vietnam, and Laos within Asia (Kuo et al., 2017; Zhang et al., 2023). The predicted distribution of *F. tikoua* in China aligns closely with that described by Deng et al. (2020), corroborating the model’s validity. Peripheral zones adjacent to core distribution areas were classified as moderately or marginally suitable habitats, with the potential to transition into highly suitable regions under future climates. In the present study, two socio-economic paths, SSP1-2.6 and SSP5-8.5, were considered. Among them, SSP1-2.6 represents a lower greenhouse gas emission level and relatively small climate change, consistent with the temperature rise not exceeding 2 °C by 2100 (Zhao et al., 2024). SSP5-8.5 is a development path based on the continued elevated use of fossil fuels, representing high greenhouse gas emissions (Xu et al., 2024; Zhang et al., 2019). Therefore, there is a greater degree of climate warming under the SSP5-8.5 scenario relative to the SSP1-2.6 scenario. The analysis of the distribution of potential suitable areas of the two species in the future climate scenarios showed that the potential distribution areas of the two plant species are likely to expand under both scenarios. Species unable to adapt rapidly to climate change face the risk of distributional contraction and reduced genetic diversity (Gidden et al., 2019; Lyons et al., 2010; Usta et al., 2022). The superior environmental adaptability and stress resistance of the two plant species support their continuous expansion under future climate change; this also reflects that *P. tricuspidata* and *F. tikoua* are promising plant species for both future ecological protection and environmental remediation.

### 4.2 Factors driving the distribution of *P. tricuspidata* and *F. tikoua*

Pearson correlation analysis and contribution degree should be used to effectively screen environmental variables in order to make reliable predictions of potential suitable areas (Shi et al., 2024). In the present study, environmental variables showed strong contributions to the distribution of both species. Temperature seasonality was the primary factor influencing the potential distribution of *P. tricuspidata* (26.3%), with higher suitability observed at values ranging from approximately 716.531 to 2579.135, suggesting an adaptation to environments with pronounced annual temperature fluctuations. In contrast, precipitation of the warmest quarter exerted the greatest influence on *F. tikoua* (35.7%), with favorable conditions occurring between about 464.736 and 2719.200 mm, highlighting the importance of water availability during the growing season for this species. The distributions of the two liana species are mainly influenced by water availability and temperature. Since all ecosystems on Earth are interconnected and sustained by water, water availability is a primary driver of species distribution patterns (Gutiérrez-Hernández and García, 2021). In addition, each plant species has an optimal temperature range for growth, highlighting temperature as a crucial climatic factor determining plant growth and productivity (Chaudhary et al., 2019). Therefore, precipitation and temperature act as key environmental factors controlling the ecological processes and potential distributions of *P. tricuspidata* and *F. tikoua*. The contribution analysis also showed that the importance of pedological variables differed between the two species. For *P. tricuspidata*, the dominant predictors were mainly climatic, and the retained soil variables contributed only marginally to the final model. In contrast, for *F. tikoua*, available water capacity class and other substrate-related variables made non-negligible contributions, indicating that edaphic water-holding conditions may be more important for this species at the present modelling scale. This result suggests that the added value of soil variables in this study lies primarily in improving ecological interpretation and revealing interspecific differences in environmental dependence, rather than in uniformly increasing predictor importance across both models. Although precipitation and temperature constrain their potential distributions, both species exhibit strong adaptive strategies. *P. tricuspidata* can effectively adapt to water-limited environments by regulating biomass allocation, nutrient uptake, and nutrient use efficiency (Wang et al., 2009), whereas *F. tikoua* is capable of climbing on rocks or tree trunks and shows strong tolerance to extreme environmental conditions, including drought (Gong et al., 2022). These adaptive traits enable both species to survive and expand in rocky slope habitats characterized by poor soils and limited water availability, indicating their considerable potential for application in rocky slope ecological restoration.

### 4.3 Adaptation of *P. tricuspidata* and *F. tikoua* to rocky slopes

Karst is found all over the world, including Alaska, Hawaii, Puerto Rico, the U.S. Virgin Islands, and the contiguous United States (Weary and Doctor, 2016); Central Europe (Telbisz and Mari, 2020); Southeast Australia (Osborne and Branagan, 1988); and Southeast Asia (Goldscheider et al., 2020; Tuyet, 2001), which include the main karst distribution areas. The predicted suitable habitats of *P. tricuspidata* and *F. tikoua* in our study were generally consistent with the karst distribution areas globally (Hollingsworth, 2008; Telbisz and Mari, 2020; Weary and Doctor, 2016). Infrastructure construction in these areas can very easily lead to the creation of rocky slopes, resulting in poor stability of land surfaces. The surface of karst is generally weathered, with a characteristic relative lack of arable land and nutrients that support plant growth (Cao et al., 2021; Li et al., 2020). More importantly, these factors lead to the reduction of the water storage capacities of rocky slopes. These traits make both species ecologically interesting candidates for rocky-slope rehabilitation, but they do not eliminate the need for site-level screening. The success of rocky-slope rehabilitation is strongly influenced by real-world factors. An additional issue requiring explicit attention is ecological risk in non-native regions. Rocky habitats often support relatively simple plant assemblages and may provide limited biotic resistance. Under such conditions, promoting a climatically suitable non-native climbing species could create a risk of excessive dominance or invasive spread, especially if future climate change further increases climatic suitability. This concern is particularly relevant for *P. tricuspidata*, which has already been introduced and established outside part of its native range. Therefore, increased suitability in non-native regions should be interpreted as a potential ecological warning signal as well as a possible management opportunity, and any practical introduction should be preceded by formal invasion-risk assessment and long-term ecological monitoring.

Furthermore, they can seriously hinder the survival of natural and artificially introduced vegetation. Native plants with stronger drought resistance are often selected for the ecological rehabilitation of rocky slopes. The stem growth, internodes, and petioles of *P. tricuspidata* were limited under limited availability of soil water, with accompanying enhanced root growth (Wang et al., 2010). This phenomenon is not only conducive to water accumulation but also to the stability of rocky slopes. In addition, its special metabolic regulation ability also enables *P. tricuspidata* to rapidly survive in water-deficient environments of rocky slopes. Potassium concentrations in *P. tricuspidata* tissues are usually less than 10 mg g^-1^, limiting stomatal openings and, consequently, reducing water losses (Wang et al., 2009). In contrast with *P. tricuspidata*, *F. tikoua* can effectively adapt to water shortage conditions through its unique reproduction mode. Its aerial roots are conducive to the continuous growth of small leaves of *F. tikoua* in water- and nutrient-rich areas. In addition, *P. tricuspidata* and *F. tikoua* have common features, such as their high growth rate, long growth cycle, rapid formation of vegetation canopies, and tolerance to poor environmental conditions (Liu et al., 2021; Pérez et al., 2021; Yang et al., 2018; Zhou et al., 2022), which enhances the adaptability of these plant species to the water shortage conditions that are characteristic of rocky slopes. This further supports our proposal of applying these two species to ecological rehabilitation projects of rocky slopes.

### 4.4 Strategies and suitable areas for the combined use of *P. tricuspidata* and *F. tikoua* to restore rocky slopes

As a response to the climate and environment, species will migrate according to changes in the environment. As the climate continually warms, many plant species will gradually migrate to higher altitudes or higher latitudes (Bertrand et al., 2011). For instance, the habitats of *Trichosanthes rubriflos, Trichosanthes rosthornii, and Trichosanthes kirilowii* are predicted to shift to higher elevations in mountainous areas in the future (Wang et al., 2026). Under climate change scenarios, both *P. tricuspidata* and *F. tikoua* are anticipated to extend their suitable habitats to higher-altitude karst terrains characterized by rocky slopes. Such range expansions could contribute to ecological restoration of degraded rocky slopes in these regions. *P. tricuspidata* and *F. tikoua* have different growth and morphological characteristics. *P. tricuspidata* has adhesive discs and strongly stretched tendrils, allowing it to climb smooth walls and exposed rocks. The roots of *F. tikoua* can develop along rock crevices and adhere to rock surfaces, allowing single or multiple individuals to form vegetative patches of approximately 10 to 20 m^2^. These ecological traits, together with the climbing characteristics of *P. tricuspidata*, indicate that both species are well adapted to mountainous environments and rocky substrates, including the upper layers of exposed rock faces. Based on these contrasting traits, the two species may play different but potentially complementary roles in rocky-slope revegetation. Accordingly, *P. tricuspidata* and *F. tikoua* could be strategically applied to the lower and upper sections of rocky slopes, respectively, to facilitate integrated ecological rehabilitation. More cautiously, our results suggest that the two species may be considered as complementary candidate species in broad regions where climatic suitability is favorable, subject to further verification of local rocky-slope occurrence, substrate conditions, slope aspect, hydrological setting, and ecological safety. Thus, the present study does not directly define implementation sites or engineering planting schemes; instead, it provides a first-step climate-informed screening basis for subsequent regional case studies and field validation. From a management perspective, a more realistic application pathway would involve three stages: first, identifying broad climatically suitable regions using SDM outputs; second, intersecting these regions with actual karst or rocky-slope habitats and disturbance zones; and third, conducting site-level assessments and pilot trials before implementation. In this sense, the present study is most useful as an initial regional screening exercise within a larger restoration planning workflow.

## 5 Conclusion

In summary, *P*. *tricuspidata* and *F*. *tikoua* demonstrate substantial potential for environmental remediation applications owing to its unique physiological and reproductive capabilities, which enable them to thrive in the arid, rocky slope environments of karst regions. Using an optimized MaxEnt model, we predicted the potential distributions of both species under current and future climate scenarios. At the broad spatial scale considered here, climatically suitable regions for the two species were identified in several parts of the world, including areas that contain major karst landscapes. However, these results represent broad-scale climatic suitability rather than direct identification of restoration implementation sites. Temperature seasonality and precipitation of the warmest were identified as the dominant predictors of the potential distributions of *P. tricuspidata* and *F. tikoua*, respectively. In the future, as climate anomalies intensify, the potential distribution area of both *P. tricuspidata* and *F. tikoua* is expected to increase. More precisely, the selected future scenarios suggest an overall expansion or spatial redistribution of climatically suitable areas for both species, although the magnitude and direction of change vary among scenarios and between species.Their suitable habitat is projected to include higher-elevation karst regions. However, prior to introduction, a thorough ecological risk assessment must be conducted to ensure no adverse ecosystem impacts. This precaution is particularly important in non-native regions, where increased climatic suitability may also imply elevated invasion risk. If deemed ecologically safe, these species could contribute to the restoration of degraded rocky slopes in such areas. Therefore, the present study supports the potential value of these two lianas as broad-scale candidate species for further screening in rocky-slope ecological rehabilitation. But their practical application can only be determined after integrating climatic suitability with real-world restoration constraints. Therefore, a differentiated use of the two species is suggested, with *P. tricuspidata* planted on lower slope sections and *F. tikoua* on upper slope sections. This framework can serve as a conceptual basis for future regional case studies and field testing in rocky-slope ecological rehabilitation.

## Data Availability

The original contributions presented in the study are included in the article/**Supplementary Material**. Further inquiries can be directed to the corresponding authors.
