# Supplementary material for: Climatic suitability screening of Parthenocissus tricuspidata and Ficus tikoua as candidate lianas for rocky slope rehabilitation
Source: Front Plant Sci. 2026 Apr 13;17:1790833. doi: 10.3389/fpls.2026.1790833 (PMC13111261; doi:10.3389/fpls.2026.1790833)
Supplement: Supplementary file 1 [file DataSheet1.pdf]

Supplementary Material

1 Supplementary Tables

Table S1. Contribution rates of the 28 environmental variables to the MaxEnt-based predicted results

P. tricuspidata			*F. tikoua*		
**Variable**	**Percent contribution**	**Permutation importance**	**Variable**	**Percent contribution**	**Permutation importance**
bio17	25.1	1.6	bio18	46.7	1.8
bio12	15	2.6	bio4	11.8	12.5
bio1	14.8	56.5	bio15	10.9	5.9
bio11	11.6	0.9	bio6	7.7	0.3
bio4	11.3	7.4	bio11	6.8	2.3
bio14	9.3	1.3	T_ECE	3.8	2.1
T_ECE	3.1	0.9	bio9	2.1	47.3
bio3	2.4	4.4	T_CASO4	2.1	4
bio9	1.6	0	bio19	1.7	0.9
bio2	1.4	7.6	T_CLAY	1.5	0.9
T_GRAVEL	1	1.4	bio7	1.2	0.6
bio5	0.8	1.5	REF_DEPTH	0.7	0.6
bio10	0.4	0.2	bio2	0.5	1.8
bio6	0.4	2.2	T_GRAVEL	0.5	0.2
bio13	0.3	5.1	AWC_CLASS	0.3	0.8
bio15	0.3	0.4	bio1	0.3	1.3
bio16	0.3	0.1	bio16	0.3	1.6
bio18	0.2	0.9	T_PH_H2O	0.3	0.8
bio7	0.2	4.8	bio3	0.2	1.2
bio19	0.1	0.1	bio8	0.2	1
T_CLAY	0.1	0.2	T_CACO3	0.2	0.5
AWC_CLASS	0	0	bio12	0.1	1.4
bio8	0	0	bio13	0.1	0.9
T_CACO3	0	0	bio17	0.1	7.9
REF_DEPTH	0	0	bio10	0	0.3
T_CASO4	0	0	bio14	0	0.4
T_OC	0	0	bio5	0	0.7
T_PH_H2O	0	0	T_OC	0	0.1

Table S2 Evaluation metrics of MaxEnt model generated by kuenm and optimal model parameter configuration.

Species	Model	Mean_AUC_ratio	pval_pROC	Omission_rate_at_5%	AICc	delta_AICc	W_AICc	num_parameters
Parthenocissus tricuspidata	M_0.8_F_LP	1.822	0	0.048	69408.272	0	1	23
Ficus tikoua	M_1.7_F_QTH	1.970	0	0.2	3328.8936	0	0.001	35
